# s-CAPE trauma recovery program: the need for a holistic, trauma- and violence-informed domestic violence framework

**DOI:** 10.3389/fgwh.2024.1404599

**Published:** 2024-11-07

**Authors:** Karen Williams, Merrylord Harb, Lata Satyen, Mia Davies

**Affiliations:** ^1^ Ramsay Clinic Thirroul, Wollongong, NSW, Australia; ^2^ School of Psychology, Deakin University, Burwood Campus, Burwood, VIC, Australia

**Keywords:** domestic violence, trauma, recovery, holistic, treatment

## Abstract

**Introduction:**

Domestic violence is a worldwide issue of significant concern due to its high global prevalence rates, societal costs, and the pervasive individual-level effects on physical, mental, economic, and social health and functioning. There is therefore an urgent need to deliver effective and consistent recovery services in order to mitigate the harmful societal and individual-level impacts of experiencing domestic violence and to promote victim-survivor recovery and wellbeing. This paper reviews the literature around practice models and frameworks for recovery after domestic violence and proposes the need for a holistic, trauma- and violence-informed approach to facilitate recovery and healing.

**Findings:**

Research indicates that formal supports improve recovery and wellbeing outcomes for victim-survivors, despite some literature gaps. Trauma-and-violence-informed approaches to care and holistic support are proposed as a means of improving recovery services and minimising harm to victim-survivors while maximising wellbeing. However, the literature reveals existing service gaps, including a lack of trauma-informed services and service providers engaging in practices that are retraumatising for victim-survivors.

**Discussion:**

The review findings indicate the lack of a clear and consistent evidence-based recovery framework to provide holistic, trauma-and-violence-informed care that is tailored to the needs of victim-survivors of domestic violence.

**Conclusion:**

We present the development of the s-CAPE trauma and recovery program, an integrated, holistic, trauma-and-violence-informed framework for recovery after domestic violence. s-CAPE was developed in Australia's first holistic, women's-only trauma treatment facility and is evidence-based and designed to address current service gaps, promoting positive recovery outcomes for victim-survivors.

## 1 Introduction

Domestic violence is a global epidemic, with prevalence data indicating that 30% of women worldwide, or one in three women, have experienced physical and/or sexual violence in their lifetime by an intimate partner or other family member (1, 2). The term “domestic violence” is often used interchangeably with “intimate partner violence” or “family violence”. The concept refers to controlling, threatening, or coercive behaviour intended to cause harm or suffering, perpetrated by a current or former intimate partner, or another family member or relative; the violence can be physical, sexual, psychological, emotional, financial, or spiritual in nature (3, 4). Domestic violence perpetrators tend to engage in a combination of these types of violent behaviour (5). The World Health Organization (WHO) recognizes domestic violence as a form of violence against women, with 38% of murders of women committed by intimate partners (2). This is despite concerted efforts made by international bodies, service providers, specialist practitioners, survivor advocates, policy makers, and researchers aiming to reduce the violence.

The health consequences of domestic violence are pervasive, affecting victim-survivors’ short-term and long-term wellbeing and prognosis for recovery, impacting their sexual health, and increasing their likelihood of miscarriage, traumatic brain injuries, depression, post-traumatic stress disorder (PTSD), anxiety disorders, pain syndromes, eating disorders, and suicide attempts (6–12). A combination of physical, sexual, and psychological violence has been shown to have the strongest association with mental health difficulties, including depression and PTSD (5, 13). The social consequences affect victim-survivor's ability to live safely due to homelessness, isolation resulting from stigma, loss of employment, and negative impacts on educational attainment, earnings, and both theirs and their children's ability to meaningfully re-integrate into society (14–18).

In terms of the economic cost, data are available from few countries. According to the United Nations, the economic cost of violence against women globally was estimated to be 1.5 trillion US dollars in 2016 (19). The financial cost of violence against women and their children in Australia in 2015–2016 was estimated to be 22 billion dollars (20). This cost was attributed to the pain and suffering caused by domestic violence and its impacts on children, the healthcare system, service consumption, loss of income tax, and welfare (20). Despite the significance of these individual and societal level impacts of domestic violence, there is a lack of clear and consistent approaches regarding treatment options for victim-survivors. Holistic, evidence-based methods to help women and children recover from domestic violence are lacking. Therefore, a practice framework to support victim-survivor recovery is urgently needed.

## 2 Method

A narrative literature review was undertaken as the extant literature in this area is limited. The search guidelines used were to explore the extant literature for any type of intervention to address recovery from domestic abuse. A search of various databases such as EBSCOHOST, Medline, PubMed etc. did not display the presence of a trauma and holistic recovery program for victim-survivors of domestic abuse. Therefore, an extended literature review or any other type of review, such as a systematic review was not warranted. Here, we present findings from the related literature to examine the importance of recovery programs after domestic abuse and theoretical frameworks that can guide the development of a holistic approach to supporting healing after domestic, family and sexual violence.

### 2.1 Review findings

#### 2.1.1 The importance of examining recovery after domestic violence

Recovery refers to the continuous process that victim-survivors undertake to heal and recover their lives and their physical, mental, and emotional health from the damage caused by domestic violence, enabling them to be “safe, healthy, and resilient and to have economic security and post-traumatic growth” (21). Post-traumatic growth refers to positive psychological changes following traumatic events, involving appreciation of life, meaning, and personal strengths (22–24). Victim-survivors of domestic violence experience PTSD at rates much higher than that of the general population, ranging from around 30% to 75% (25–28), compared to around 12% of the general population (29). Additionally, victim-survivors experience mental health symptoms with greater severity, with Complex-PTSD (CPTSD) more prevalent among victim-survivors than PTSD (30).

Domestic violence is considered more detrimental to mental health and functioning than that of non-interpersonal traumas, such as natural disasters, due to the experience of abuse perpetrated by a trusted person and the complex emotional states that arise as a result (31). As a result, individuals affected by domestic violence are particularly at-risk of retraumatization when accessing services to support their recovery (32); this is further exacerbated by the inadequacy of services, the lack of appropriate care that involves a trauma- and violence-informed framework, culturally incongruent care, and difficulties accessing available services due to financial, geographical, or other constraints.

Retraumatization harms recovery by leading to poorer mental health outcomes (33) and negatively impacting treatment adherence (34). Research has demonstrated, across a range of services, both inadequacies in meeting the needs of victim-survivors and harmful practices towards them, such as blaming, disbelieving, dismissing or denying their experiences, stigmatising, discriminating, and misdiagnosing (16, 32, 35–38). Further examination and action are therefore necessary to overcome the unique challenges associated with recovering from domestic violence, to mitigate its damaging impacts, and to address the current gaps in care.

#### 2.1.2 Factors affecting recovery after domestic violence

While recovery is an ongoing, long-term process, qualitative and quantitative research has demonstrated that recovery after domestic violence is possible (22, 39–42). The recovery journey of each individual is unique and complex and affected by a range of factors. For example, the nature of the violence experienced has been shown to impact recovery, including duration and types of violence. Exposure to multiple types of violence has been associated with poorer mental health outcomes (5, 13), with the highest incidence of mental health symptoms related to physical and/or sexual violence (43). Despite this, longitudinal research indicates that women exposed to both psychological and physical violence are more likely to experience a reduction in anxiety, depressive, and PTSD symptoms over time than women exposed to psychological violence alone (39). Additionally, longer duration of violence is associated with higher levels of depressive symptoms (43).

Social support is the factor most consistently shown to have a major influence on victim-survivor recovery (44). Quantitative research has demonstrated that social support is associated with reduced depressive, anxiety, and PTSD symptoms (39) and mediates the negative impacts of domestic violence on wellbeing (45). The importance of social support for recovery is also supported by qualitative research including victim-survivor voices (41). It has been demonstrated across cultures (46–48), and includes friendships, community, family, and new intimate partners (42, 44, 49).

In terms of formal support, qualitative research indicates that women and children affected by domestic violence who have accessed formal mental health support experience more positive recovery outcomes (15, 42) and quantitative research has suggested that psychological supports can reduce anxiety, depression, and PTSD symptoms among victim-survivors (50–52). Access to formal supports is influenced by geographic location, with the recovery trajectories of victim-survivors living in rural areas negatively impacted by the reduced availability of resources (53). Whether informal or formal, the support of others is evidently necessary for recovery from domestic violence, and recovery should not be accomplished alone.

The safety and stability provided by financial, employment, and housing security are key components of recovery, allowing victim-survivors to rebuild their lives and feel hopeful about the future. Research on victim-survivors’ perspectives has demonstrated that they consider work, education, financial security, and secure accommodation as essential to their recovery and safety (22, 40, 47, 54). Other factors suggested to facilitate recovery after domestic violence include hope (55, 56), resilience (57), self-worth (41), reducing guilt (42), self-care (41, 54), spirituality (22), parenting support (40), and helping others (58, 59). These elements should be incorporated into victim-survivor treatment programs to most effectively support recovery and healing.

#### 2.1.3 Theories of recovery

Scholars have conceptualised recovery as a process whereby victim-survivors reconnect with the self, others, and the world (60) through interaction with a number of internal and external processes (41, 61). Some researchers have framed recovery as a linear, developmental process occurring in a series of stages (62, 63), while recent research indicates that recovery after domestic violence is a complex, non-linear and multidimensional process (23, 40, 58). Recovery involves a general sense of moving in a forward direction, during which victim-survivors will cycle through a range of emotions, experiences, and challenges in different domains, with growth experiences and recovery benchmarks interwoven throughout.

Researchers have sought to identify the specific elements involved in recovery trajectories after domestic violence. Carman et al. (40) used victim-survivor definitions of recovery to determine that recovery consists of five core themes: survival and safety, gaining freedom, moving on, a better life, and children and parenting—specifically for parents. A better life, for example, refers to finding enjoyment, purpose, and appreciation of life. These themes are conceptualised as a series of interwoven threads that victim-survivors manage concurrently, while the focus and order of prioritisation of each thread changes during different life circumstances (40). Despite these general trends, researchers tend to agree that the recovery journey is multifaceted and unique to each individual (23, 40, 41, 64). Therefore, any service supporting recovery should be adapted to the unique needs of each victim-survivor.

#### 2.1.4 Frameworks for recovery

Bessel Van der Kolk's (65) acclaimed work on trauma contends that it is possible to reverse the neural damage caused by traumatic experiences through a combination of top-down (i.e., through understanding, connecting with others, and processing traumatic memories) and bottom-up (i.e., regulating and connecting with the body) approaches, and medication or other technologies to change brain functioning. He states that individuals who have experienced trauma need to “befriend the sensations in their bodies”, learn to calm themselves, and be fully present and honest with themselves about the impacts of trauma (65], p. 100). These concepts have informed treatment models for complex trauma, which focus on establishing safety through the therapeutic relationship, symptom management, and emotion regulation, recovering from the trauma using memory processing and mindfulness, and rebuilding one's life by finding purpose, meaning, and skill development (52).

Recommendations for service provision for individuals affected by domestic violence tend to build on these complex trauma models by incorporating similar elements and tailoring them to victim-survivor needs, such as emphasis on a trusting therapeutic relationship and building both emotional and physical safety. It has been proposed that safety and freedom can be encouraged by supporting victim-survivors to recognise the tactics of control employed by their perpetrator, to regulate trauma symptoms, and through processing of traumatic memories (40). Helping victim-survivors build self-worth is also considered a key component for any recovery model (60). The therapeutic relationship is particularly important to any psychological supports for victim-survivors, and recommendations emphasise empathy, respect, and supportive counselling (40, 47, 64). Practitioners can support victim-survivors to establish healthy boundaries, develop positive relationships, identify their strengths and resources, build skills, and provide practical support to manage the demands of life (40, 42, 44). Practitioners are encouraged to provide a non-blaming environment and to recognize the survival and coping abilities of victim-survivors (44).

Formal support has been positively associated with victim-survivor recovery, yet, while some researchers have proposed specific frameworks for facilitating recovery after domestic violence, these are limited. Sullivan (64) proposed the Social and Emotional Well-Being Framework as guiding the practices of different domestic violence services. This framework indicates that services aim to help victim-survivors to thrive and emphasize community and social supports in the recovery process. Essential components of this framework include providing information to victim-survivors, safety planning, skill-building, offering empathy and encouragement, counselling, increasing access to resources and opportunities, social support, and promoting community/systems change (64).

In terms of mental health support, review papers have shown that psychological interventions can reduce depression, anxiety, and PTSD symptoms in women victim-survivors; however, the authors expressed concerns over methodological limitations (51, 52). However, a Cochrane review deemed it unclear whether psychological interventions can reduce PTSD symptoms in victim-survivors due to a lack of evidence (66). One review found that effective mental health interventions were typically cognitive-based or cognitive behavioural therapy (CBT), typically including problem-solving and cognitive techniques to alter distorted thoughts about the self (51). Therapies that have been adapted to be victim-survivor specific have shown some promise, despite research concluding that the evidence base is minimal (52). One example is the Cognitive Trauma Therapy for Battered Women modified CBT program which incorporates victim-survivor specific elements into CBT, such as addressing risk, ongoing contact with perpetrators, and guilt (67).

Despite the existence of victim-survivor-specific interventions, the evidence base is lacking, the approaches and theoretical frameworks used tend to be heterogeneous and inconsistent (51), while the therapies are narrow in scope and not necessarily trauma-informed or addressing the holistic needs of victim-survivors. Furthermore, a practice gap exists in that victim-survivors continue to report negative experiences when accessing services for their recovery, emphasising the need for a consistent recovery framework and practice model that addresses victim-survivor needs.

## 3 Discussion

The findings of this mini-review demonstrate the severity of the issue of domestic violence at individual, societal, and global levels. Domestic violence is highly prevalent, considered a global problem of epidemic proportions (2), and associated with significant costs at the societal level. The individual impacts of experiencing domestic violence are long-term, pervasive, and detrimental to all dimensions of health, including physical, mental, economic, and social health and functioning. Despite this, recovery after domestic violence is possible. Recovery is influenced by a range of factors including the nature of the violence, social support, access to formal supports, and housing, economic, and occupational security. Access to formal supports is essential to victim-survivor recovery and wellbeing, promoting improvements in their mental health and overall functioning. However, clear and consistent practice guidelines and frameworks for supporting victim-survivor recovery and healing are lacking, and victim-survivors continue to be retraumatized when accessing services, with services failing to meet their needs or engaging in harmful practices towards them. Therefore, clear and consistent, evidence-based recovery frameworks are urgently needed to mitigate the damaging effects of domestic violence and support victim-survivor recovery, health, and wellbeing.

Trauma-informed care is an approach to service provision aiming to minimize harm and increase positive outcomes by engaging in practices that incorporate an awareness and sensitivity to the effects of trauma on individuals accessing services (68). When practitioners become aware of the impacts of trauma and how these might impact victim-survivor service engagement, services can better support recovery and healing, reducing the gap between service delivery and the needs of victim-survivors (69). Trauma-informed practices reduce the possibility for retraumatization and have been linked to better care experiences, improved mental health outcomes, and increased service utilization (70–74).

Trauma-and-violence-informed care extends the concept of trauma-informed care through incorporating an awareness of the contexts and structures in which violence is embedded, for both victimisation and perpetration. Contextual and structural factors include social and economic inequalities, discrimination, historical experiences of violence, and systemic violence (75). This framework expands the conceptualisation of violence from placing focus on the individual to exploring the broader social, cultural, historical, and political contexts within which violence occurs (75). Research demonstrates that this conceptualisation allows greater understanding of the challenges that victim-survivors face and therefore services that use trauma-and-violence-informed care are better placed to comprehensively address these challenges. This framework emphasises the importance of considering intersectionality, or the interaction of different dimensions of inequality and male violence, when working with victim-survivors (76, 77). This lens is particularly important when working with victim-survivors from culturally diverse backgrounds, migrants, or those experiencing homelessness. For example, one study highlighted the gender inequality and structural violence implicated in female homelessness (78) while Satyen et al. (48) have reviewed the importance of culturally appropriate care for people from multicultural and minority backgrounds.

As discussed previously, the recovery process is complex, and victim-survivors have a range of needs across different areas. This is indicated by the domestic violence sector encompassing a vast system of services including primary healthcare, social, legal, and community services, drug and alcohol, psychiatric, non-government organizations, housing, advocacy, and the police. Recovery frameworks should therefore incorporate holistic approaches to care to comprehensively meet the needs of victim-survivors in their recovery. Sexual violence research has indicated that the use of holistic services enhances the healing of women affected by sexual violence (79). Service integration, or combining multiple services into one, has been proposed as a means of providing more holistic trauma-informed care (80). The use of trauma-informed care is therefore highly beneficial in supporting the recovery of victim-survivors when accessing services and is thus a central focus of the s-CAPE trauma and recovery program.

The s-CAPE (escape) trauma and recovery program is an integrated, holistic, trauma and violence-informed framework for recovery, developed at the Ramsay Clinic Thirroul in Australia to address the lack of programs addressing recovery and healing of victim-survivors of domestic violence. The model is evidence-based, aiming to encapsulate all aspects identified by the research as effective in addressing trauma. The s-CAPE program commenced in 2022 in Australia's first women's-only, trauma-specific hospital. The program supports victim-survivors of domestic and sexual violence to escape the violence, the perpetrators, the injury of trauma, the shame, the self-loathing, and other complex post-traumatic stress symptoms associated with the abuse. It is a comprehensive program that focuses on safety, community, attachment, processing, and empowerment and is delivered by trauma-and-violence-informed specialist mental health practitioners.

## 4 Conclusion

This review has asserted the urgent need for a woman-centred, trauma-and-violence-informed care model for people impacted by domestic violence and proposed a new model of care in the s-CAPE trauma and recovery program. The extant literature revealed the absence of any framework in the world incorporating a holistic, trauma-and-violence-informed approach for the specific care of domestic violence survivors. In practice, we are aware of the Ramsay Clinic Thirroul in New South Wales, Australia, as Australia's first women's only, stand-alone, trauma informed mental health facility that provides specialized care for people with trauma-related mental health disorders, including women who have been subjected to domestic violence. This facility implements a holistic approach incorporating the treatment of mental, physical, nutritional, and social health of women survivors. The impact of such a framework on various aspects of women's mental, physical and social health and the resulting cost effectiveness to the health care system needs to be examined in detail. A careful analysis and evaluation of this and any other such practices will inform other domestic violence services of the framework required to support women and children affected by domestic violence in their recovery and healing journey.

## References

[1] Sardinha L Maheu-Giroux M Stöckl H Meyer SR García- Moreno C. Global, regional, and national prevalence estimates of physical or sexual, or both, intimate partner violence against women in 2018. Lancet. (2022) 399(10327):803–13. 10.1016/S0140-6736(21)02664-7 35182472 PMC8885817

[2] World Health Organization. Violence Against Women. Geneva: World Health Organisation (2024). Available online at: https://www.who.int/news-room/fact-sheets/detail/violence-against-women

[3] Stark E. Rethinking coercive control. Violence Against Women. (2009) 15(12):1509–25. 10.1177/1077801209347452 19850959

[4] Moshtagh M Amiri R Sharafi S Arab-Zozani M. Intimate partner violence in the Middle East region: a systematic review and meta-analysis. Trauma Violence Abuse. (2023) 24(2):613–31. 10.1177/15248380211036060 34382453

[5] Dutton MA Kaltman S Goodman LA Weinfurt K Vankos N. Patterns of intimate partner violence: correlates and outcomes. Violence Vict. (2005) 20(5):483–97. 10.1891/vivi.2005.20.5.483 16248486

[6] Coker AL Davis KE Arias I Desai S Sanderson M Brandt HM Physical and mental health effects of intimate partner violence for men and women. Am J Prev Med. (2002) 23(4):260–8. 10.1016/S0749-3797(02)00514-7 12406480

[7] Coker AL. Does physical intimate partner violence affect sexual health? A systematic review. Trauma Violence Abuse. (2007) 8(2):149–77. 10.1177/1524838007301162 17545572

[8] Devries KM Watts C Yoshihama M Kiss L Schraiber LB Deyessa N Violence against women is strongly associated with suicide attempts: evidence from the WHO multi-country study on women’s health and domestic violence against women. Soc Sci Med. (2011) 73(1):79–86. 10.1016/j.socscimed.2011.05.006 21676510

[9] Devries KM Mak JY Bacchus LJ Child JC Falder G Petzold M Intimate partner violence and incident depressive symptoms and suicide attempts: a systematic review of longitudinal studies. PLoS Med. (2013) 10(5):e1001439. 10.1371/journal.pmed.1001439 23671407 PMC3646718

[10] Dillon G Hussain R Loxton D Rahman S. Mental and physical health and intimate partner violence against women: a review of the literature. Int J Family Med. (2013) 2013(1):1–15. 10.1155/2013/313909 PMC3566605 23431441

[11] White M Satyen L. Cross-cultural differences in intimate partner violence and depression: a systematic review. Aggress Violent Behav Rev J. (2015) 24:120–30. 10.1016/j.avb.2015.05.005

[12] World Health Organization. Global and Regional Estimates of Violence Against Women: Prevalence and Health Effects of Intimate Partner Violence and non-partner sexual Violence. Geneva: World Health Organisation (2013).

[13] Basile KC Arias I Desai S Thompson MP. The differential association of intimate partner physical, sexual, psychological, and stalking violence and posttraumatic stress symptoms in a nationally representative sample of women. J Trauma Stress. (2004) 17(5):413–21. 10.1023/B:JOTS.0000048954.50232.d8 15633920

[14] Adams AE Greeson MR Kennedy AC Tolman RM. The effects of adolescent intimate partner violence on women’s educational attainment and earnings. J Interpers Violence. (2013) 28(17):3283–300. 10.1177/0886260513496895 23920337

[15] Green J Satyen L Toumbourou JW. Influence of cultural norms on formal service engagement among survivors of intimate partner violence: a qualitative meta-synthesis. Trauma Violence Abuse. (2023) 25(1):738–51. 10.1177/15248380231162971 37073947 PMC10666477

[16] Murvartian L Saavedra-Macías FJ Infanti JJ. Public stigma toward women victims of intimate partner violence: a systematic review. Aggress Violent Behav. (2023) 73:1349–64. 10.1016/j.avb.2023.101877

[17] Saint Arnault D O’Halloran S. Using mixed methods to understand the healing trajectory for rural Irish women years after leaving abuse. J Res Nurs. (2016) 21(5–6):369–83. 10.1177/1744987116649636

[18] Satyen L Piedra S Ranganathan A Golluccio N. Intimate partner violence and help-seeking behaviour among migrant women in Australia. J Fam Violence. (2018) 33(7):447–56. 10.1007/s10896-018-9980-5

[19] United Nations Women. The economic costs of violence against women. Remarks by UN Assistant Secretary-General and Deputy Executive Director of UN Women, Lakshmi Puri at the high-level discussion on the “Economic Cost of Violence against Women”. (2016). Available online at: https://www.unwomen.org/en/news/stories/2016/9/speech-by-lakshmi-puri-on-economic-costs-of-violence-against-women (accessed December 19, 2023).

[20] KPMG. The cost of violence against women and their children in Australia. May 2016. (2016). Available online at: https://www.dss.gov.au/sites/default/files/documents/08_2016/the_cost_of_violence_against_women_and_their_children_in_australia_-_summary_report_may_2016.pdf (accessed December 20, 2023).

[21] Department of Social Services. The National Plan to End Violence Against Women and Children 2022-2032. Commonwealth of Australia. (2022). Available online at: https://www.dss.gov.au/sites/default/files/documents/10_2023/national-plan-end-violence-against-women-and-children-2022-2032.pdf (accessed December 19, 2023).

[22] Amarsanaa K Kovács M Rácz J. Posttraumatic growth: experiences of Mongolian and Hungarian survivors of intimate partner violence. Acta Psychol (Amst). (2023) 233:1–9. 10.1016/j.actpsy.2023.103825 36630739

[23] D’Amore C Martin SL Wood K Brooks C. Themes of healing and posttraumatic growth in women survivors’ narratives of intimate partner violence. J Interpers Violence. (2021) 36(5/6):NP2697–724. 10.1177/0886260518767909 29642769

[24] Tedeschi RG Shakespeare-Finch J Taku K Calhoun LG. Posttraumatic growth: theory, research, and application. 1st ed. Rothledge. (2018):3–7. 10.4324/9781315742908-8

[25] Golding JM. Intimate partner violence as a risk factor for mental disorders: a meta-analysis. J Fam Violence. (1999) 14:99–132. 10.1023/A:1022079418229

[26] Kelly UA. Symptoms of PTSD and major depression in latinas who have experienced intimate partner violence. Issues Ment Health Nurs. (2010) 31(2):119–27. 10.3109/01612840903312020 20070226

[27] Nathanson AM Shorey RC Tirone V Rhatigan DL. The prevalence of mental health disorders in a community sample of female victims of intimate partner violence. Partner Abuse. (2012) 3(1):59–75. 10.1891/1946-6560.3.1.59 22741043 PMC3381987

[28] Pico-Alfonso MA Garcia-Linares MI Celda-Navarro N Blasco-Ros C Echeburúa E Martinez M. The impact of physical, psychological, and sexual intimate male partner violence on women’s mental health: depressive symptoms, posttraumatic stress disorder, state anxiety, and suicide. J Womens Health. (2006) 15(5):599–611. 10.1089/jwh.2006.15.599 16796487

[29] Australian Institute of Health and Welfare. Stress and trauma. Australian Government, AIHW. (2022). Available online at: https://www.aihw.gov.au/reports/mental-health/stress-and-trauma (accessed February 21, 2024).

[30] Fernández-Fillol C Pitsiakou C Perez-Garcia M Teva I Hidalgo-Ruzzante N. Complex PTSD in survivors of intimate partner violence: risk factors related to symptoms and diagnoses. Eur J Psychotraumatol. (2021) 12(1):2003616. 10.1080/20008198.2021.2003616 34925711 PMC8682852

[31] Martin CG Cromer LD DePrince AP Freyd JJ. The role of cumulative trauma, betrayal, and appraisals in understanding trauma symptomatology. Psychological Trauma. (2013) 5:110–8. 10.1037/a0025686 PMC3608140 23542882

[32] Taylor P. System entrapment: dehumanization while help-seeking for suicidality in women who have experienced intimate partner violence. Qual Health Res. (2020) 30(4):530–46. 10.1177/1049732319857671 31303117

[33] Duckworth MP Follette VM. Retraumatization: Assessment, Ttreatment, and Pprevention. New York: Routledge/Taylor & Francis Group (2012). 10.4324/9780203866320

[34] Dallam SJ. A model of the retraumatization process: a meta-synthesis of childhood sexual abuse survivors’ experiences in healthcare. Dissert Abstract Int Section B Sci Eng. (2010) 71(2-B):920.

[35] Loeb TB Joseph NT Wyatt GE Zhang M Chin D Thames A Predictors of somatic symptom severity: the role of cumulative history of trauma and adversity in a diverse community sample. Psychological trauma: theory, research. Pract Policy. (2018) 10(5):491–8. 10.1037/tra0000334 PMC6021222 29154595

[36] Marsden S Humphreys C Hegarty K. Women survivors’ accounts of seeing psychologists: harm or benefit? J Gender-Based Violence. (2021) 5(1):111–27. 10.1332/239868020X16040863370635

[37] Smith CP Freyd JJ. Insult, then injury: interpersonal and institutional betrayal linked to health and dissociation. J Aggress Maltreat Trauma. (2017) 26:1–15. 10.1080/10926771.2017.1322654

[38] Toccalino D Haag H Estrella MJ Cowle S Fuselli P Ellis MJ The intersection of intimate partner violence and traumatic brain injury: findings from an emergency summit addressing system-level changes to better support women survivors. J Head Trauma Rehab. (2022) 37(1):E20–9. 10.1097/HTR.0000000000000743 34985037

[39] Blasco-Ros C Sanchez-Lorente S Martinez M. Recovery from depressive symptoms, state anxiety and post-traumatic stress disorder in women exposed to physical and psychological, but not to psychological intimate partner violence alone: a longitudinal study. BMC Psychiatry. (2010) 10:98. 10.1186/1471-244X-10-98 21108834 PMC3009625

[40] Carman MJ Kay-Lambkin F Burgman I. Long-Term recovery from intimate partner violence: definitions by Australian women. J Fam Violence. (2023) 38(4):747–60. 10.1007/s10896-022-00389-3

[41] Flasch P Fall K Stice B Easley R Murray C Crowe A. Messages to new survivors by Longer-term survivors of intimate partner violence. J Fam Violence. (2020) 35(1):29–42. 10.1007/s10896-019-00078-8

[42] Katz E. Recovery-Promoters: ways in which children and mothers support one another’s recoveries from domestic violence. Br J Soc Work. (2015) 45:i153–69. 10.1093/bjsw/bcv091

[43] Bonomi AE Thompson RS Anderson ML Reid RJ Carrell D Dimer JA Intimate partner violence and women’s physical, mental, and social functioning. Am J Prev Med. (2006) 30:458–66. 10.1016/j.amepre.2006.01.015 16704938

[44] Alcantud PM Campdepadrós-Cullell R Fuentes-Pumarola C Mut-Montalvà E. ‘I think I will need help’: a systematic review of who facilitates the recovery from gender-based violence and how they do so. Health Expect. (2021) 24(1):1–7. 10.1111/hex.13157 PMC7879545 33216430

[45] Beeble ML Bybee D Sullivan CM Adams AE. Main, mediating, and moderating effects of social support on the well- being of survivors of intimate partner violence across 2 years. J Consult Clin Psychol. (2009) 77(4):718–29. 10.1037/a0016140 19634964

[46] Meyer S Stambe R. Indigenous women’s experiences of domestic and family violence, help-seeking and recovery in regional Queensland. Aust J Soc Issue (John Wiley & Sons, Inc.). (2021) 56(3):443–58. 10.1002/ajs4.128

[47] Oke M. Remaking self after domestic violence: mongolian and Australian women’s narratives of recovery. Australian New Zealand J Family Therapy. (2008) 29(3):148–55. 10.1375/anft.29.3.148

[48] Satyen L Rogic A Supol S. Intimate partner violence and help-seeking behaviour: a systematic review of cross-cultural differences. J Immigr Minor Health. (2019) 21:879–92. 10.1007/s10903-018-0803-9 30105537

[49] Flasch P Boote D Robinson EH. Considering and navigating new relationships during recovery from intimate partner violence. J Counsel Develop. (2019) 97(2):148–59. 10.1002/jcad.12246

[50] Melton H Meader N Dale H Wright K Jones-Diette J Temple M Interventions for adults with a history of complex traumatic events: the INCiTE mixed-methods systematic review. Health Technol Assess. (2020) 24(43):1–312. 10.3310/hta24430 32924926 PMC7520719

[51] Trabold N McMahon J Alsobrooks S Whitney S Mittal M. A systematic review of intimate partner violence interventions: state of the field and implications for practitioners. Trauma Violence Abuse. (2020) 21(2):311–25. 10.1177/1524838018767934 29649966

[52] Warshaw C Sullivan CM Rivera EA. A systematic review of trauma-focused interventions for domestic violence survivors. (2013). www.dvevidenceproject.org

[53] Stone R Campbell JK Kinney D Rothman EF. “He would take my shoes and all the baby’s warm winter gear so we couldn’t leave”: barriers to safety and recovery experienced by a sample of Vermont women with partner violence and opioid use disorder experiences. J Rural Health. (2021) 37(1):35–44. 10.1111/jrh.12518 32929780

[54] Hetling A Dunford A Lin S Michaelis E. Long-term housing and intimate partner violence: journeys to healing. Affilia J Women Soc Work. (2018) 33(4):526–42. 10.1177/0886109918778064

[55] Carman MJ Kay-Lambkin F. Long-Term recovery from intimate partner violence: recovery and hope. Int J Environ Res Public Health. (2022) 19:21. 10.3390/ijerph192113825 PMC9654800 36360705

[56] Schrank B Bird V Rudnick A Slade M. Determinants, self-management strategies and interventions for hope in people with mental disorders: systematic search and narrative review. Soc Sci Med. (2012) 74:554–64. 10.1016/j.socscimed.2011.11.008 22240450

[57] Song L. Service utilization, perceived changes of self, and life satisfaction among women who experienced intimate partner abuse: the mediation effect of empowerment. J Interpers Violence. (2012) 27:1112–36. 10.1177/0886260511424495 22203616

[58] Flasch PS Murray CE Crowe A. Overcoming abuse: a phenomenological investigation of the journey to recovery from past intimate partner violence. J Interpers Violence. (2015) 32(22):3373–401. 10.1177/0886260515599161 26261234

[59] Murray CE King K Crowe A Flasch PS. Survivors of intimate partner violence as advocates for social change. J Soc Act Counsel Psychol. (2015) 7(1):84–100. 10.33043/JSACP.7.1.84-100

[60] Sinko L Saint Arnault D. Finding the strength to heal: understanding recovery after gender-based violence. Violence Against Women. (2020) 26(12-13):1616–35. 10.1177/1077801219885185 31707935

[61] Davis K Taylor B. Stories of resistance and healing in the process of leaving abusive relationships. Contemp Nurse. (2006) 21(2):199–208. 10.5172/conu.2006.21.2.199 16696602

[62] Laing L Humphreys C. Social Work and Domestic Violence: Developing Critical and Reflective Practice. London: SAGE (2014).

[63] Wuest J Merritt-Gray M. Beyond survival: reclaiming self after leaving an abusive male partner. Can J Nurs Res. (2001) 32:79–94. 11928303

[64] Sullivan CM. Understanding how domestic violence support services promote survivor well-being: a conceptual model. J Fam Violence. (2018) 33(2):123–31. 10.1007/s10896-017-9931-6 29367804 PMC5760592

[65] Van der Kolk BA. The Body Keeps the Score: Mind, Brain and Body in the Healing of Trauma/Bessel van der Kolk. New York: Penguin Books (2015).

[66] Hameed M O'Doherty L Gilchrist G Tirado-Muñoz J Taft A Chondros P Psychological therapies for women who experience intimate partner violence. Cochrane Database Syst Rev. (2020) 2020(7):CD013017. 10.1002/14651858.CD013017.pub2 32608505 PMC7390063

[67] Kubany ES Hill EE Owens JA. Cognitive trauma therapy for battered women with PTSD: preliminary findings. J Trauma Stress. (2003) 16(1):81–91. 10.1023/A:1022019629803 12602656

[68] Substance Abuse and Mental Health Services Administration (SAMHSA). Trauma- informed approach and trauma-specific interventions. U.S. Department of Health & Human Services. (2017). Available online at: https://www.samsha.gov/nctic/trauma-interventions (accessed December 19, 2023).

[69] Kulkarni S. Intersectional trauma-informed intimate partner violence (IPV) services: narrowing the gap between IPV service delivery and survivor needs. J Fam Violence. (2019) 34(1):55–64. 10.1007/s10896-018-0001-5

[70] Edwards KM Murphy S Palmer KM Chapo S Ekdahl BA Haynes EE Co-occurrence of and recovery from substance abuse and lifespan victimization: a qualitative study of female residents in trauma-informed sober living homes. J Psychoact Drugs. (2017) 49(1):74–82. 10.1080/02791072.2016.1273566 28085637

[71] Gilbert AR Domino ME Morrissey JP Gaynes BN. Differential service utilization associated with trauma-informed integrated treatment for women with co-occurring disorders. Adm Policy Ment Health. (2012) 39(6):426–39. 10.1007/s10488-011-0362-z 21706408 PMC3652378

[72] Green BL Saunders PA Power E Dass-Brailsford P Schelbert KB Giller E Trauma-informed medical care: patient response to a primary care provider communication training. J Loss Trauma. (2016) 21(2):147–59. 10.1080/15325024.2015.1084854 27721673 PMC5051697

[73] Lietz CA Lacasse JR Cheung JR. A case study approach to mental health recovery: understanding the importance of trauma-informed care. Ethic Hum Psychol Psychiat Int J Crit Inquir. (2014) 16(3):167–82. 10.1891/1559-4343.16.3.167

[74] Morrissey JP Jackson EW Ellis AR Amaro H Brown VB Najavits LM. Twelve-month outcomes of trauma-informed interventions for women with co-occurring disorders. Psychiatr Serv. (2005) 56:1213–22. 10.1176/appi.ps.56.10.1213 16215186

[75] Wathen CN Mantler T. Trauma- and violence-informed care: orienting intimate partner violence interventions to equity. Curr Epidemiol Rep. (2022) 9(4):233–44. 10.1007/s40471-022-00307-7 36212738 PMC9527731

[76] Crenshaw K. Mapping the margins: intersectionality, identity politics, and violence against women of color. Stanford Law Rev. (1991) 43(6):1241–99. 10.2307/1229039

[77] Sweet PL. The paradox of legibility: domestic violence and institutional survivorhood. Soc Probl. (2019) 66(3):411–27. 10.1093/socpro/spy012

[78] Milaney K Lockerbie SL Fang XY Ramage K. The role of structural violence in family homelessness. Can J Pub Health/Revue Canadienne de Santé Publique. (2019) 110(5):554–62. 10.17269/s41997-019-00219-y 31077070 PMC6964589

[79] Hegarty K Tarzia L Fooks A Rees S. Women’s input into a trauma-informed systems model of care in health settings (the WITH study): key findings and future directions. Res Policy Pract. (2017) 2:3. ANROWS.

[80] Mantler T Wolfe B. Evaluation of trauma-informed integrated health models of care for women: a qualitative case study approach. Partner Abuse. (2018) 9:118–36. 10.1891/1946-6560.9.2.118

